# Targeting desmoplasia in pancreatic cancer as an essential first step to effective therapy

**DOI:** 10.18632/oncotarget.27745

**Published:** 2020-09-22

**Authors:** Nancy D. Ebelt, Vic Zamloot, Edwin R. Manuel

**Affiliations:** ^1^Department of Immuno-Oncology, Beckman Research Institute of the City of Hope, Duarte, CA 91010, USA

**Keywords:** pancreatic cancer, desmoplasia, fibrosis, hyaluronan, collagen

## Abstract

Pancreatic cancer is considered one of the most lethal cancers in the US. It contributes to an estimated 47,000 deaths annually and is predicted to surpass prostate, breast and colorectal cancers as the leading cause of cancer-related death. Although major advancements in cancer treatment have improved outcomes for many cancer types, survival rate for pancreatic cancer has not improved in nearly four decades despite tremendous effort. One attribute of pancreatic cancer that is considered a major barrier to effective treatment is the formation of fibrotic tissue around tumor cells known as desmoplasia. A number of promising approaches have been developed to deplete fibrotic components in pancreatic tumors to enhance drug delivery, some of which have been tested in clinical trials of advanced, unresectable pancreatic cancer. Here, we discuss previous efforts, shortcomings and new considerations for developing more effective agents to eliminate desmoplasia.

## INTRODUCTION

**Figure 1:**
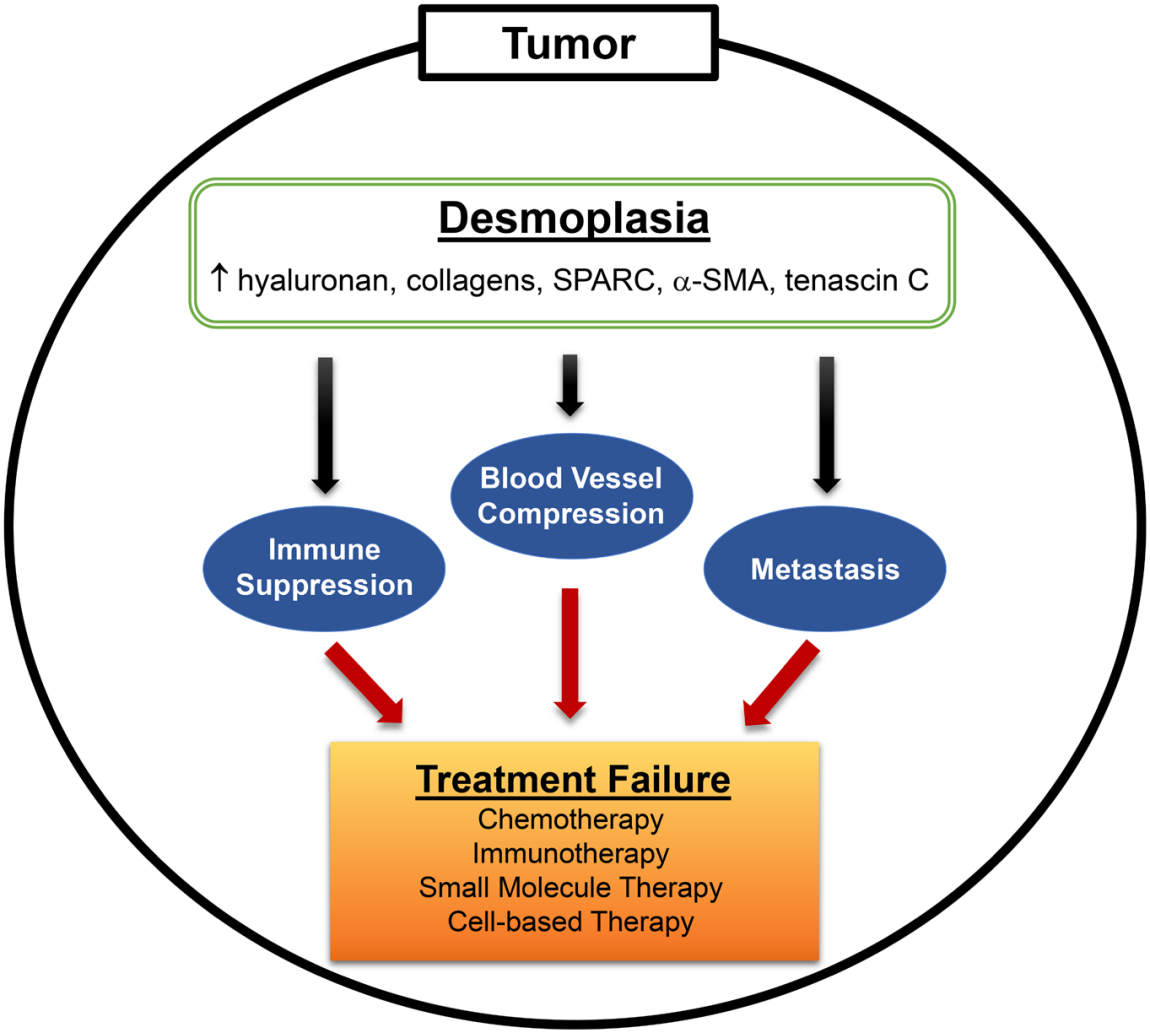
Desmoplasia is a major hallmark of PDAC. Desmoplasia, or the desmoplastic reaction, is characterized by the prevalent growth of dense fibrotic tissue around tumor cells. Activated stromal cells (fibroblasts, myofibroblasts, stellate cells) and, in some cases, tumor cells overexpress various extracellular matrix (ECM) components (hyaluronan, collagen, SPARC, α-SMA, tenascin C) that contribute to fibrosis. Hyaluronan and collagen (predominantly types I and IV) contribute to the bulk of interstitial forces that leads to blood vessel compression, while also acting as ligands for immune suppressive signaling in T and NK cells. Constant remodeling of the tumor stroma, involving breakdown and synthesis of ECM components, also facilitates distal metastasis. Overall, the presence of desmoplasia in PDAC impedes anti-cancer drug efficacy leading to treatment failure and, thus, serves as an important target that should be eliminated prior to therapy.

Pancreatic cancer is currently the third leading cause of cancer-related death in the United States and is projected to become the second leading cause by 2030. Nearly 95% of pancreatic cancers develop in exocrine tissue and are predominantly pancreatic ductal adenocarcinomas (PDAC). PDAC progresses slowly and is often diagnosed at late stage in > 50% of patients, which is associated with a meager 5-year survival rate of only ~3%. Whereas major advancements in cancer therapy have drastically improved overall patient survival for various cancer types, survival rates for PDAC have not changed significantly in nearly four decades. PDAC remains refractory to many administered therapies due to one major hallmark: desmoplasia (Figure 1). Desmoplasia is the formation of dense fibrotic tissue within and around tumor tissue, which is generated by activated fibroblasts, myofibroblasts, pancreatic stellate cells and tumor cells, and comprises 80% to 90% of the tumor volume [1]. The fibrotic tissue is composed of an overabundance of extracellular matrix (ECM) components, which includes hyaluronan, collagens, α-smooth muscle actin (α-SMA), and tenascin C, and the matricellular protein secreted protein acidic and rich in cysteine (SPARC). In addition to acting as a physical barrier, excess ECM deposition contributes to significant increases in interstitial pressure that leads to compression of blood vessels, ultimately blocking the main conduits for efficient drug delivery. Moreover, certain ECM components - such as hyaluronan, collagen, and tenascin-are known to participate in inhibitory signaling pathways that suppress anti-tumor immune responses [2]. Thus, methods that effectively eliminate desmoplasia prior to or during treatment will be more likely to improve efficacy of both chemo- and immunotherapy.

Previous strategies to target desmoplasia in PDAC have achieved mixed results. IPI-926 is a Smoothened (Smo) inhibitor that was shown to decrease stromal components in a spontaneous model of PDAC that, in turn, enhanced delivery and efficacy of chemotherapy to prolong survival [3]. However, phase II clinical trials were halted when patients receiving gemcitabine with placebo were living longer than patients receiving gemcitabine with IPI-926. Follow-up studies would later determine that Hedgehog pathway inhibition, in which Smo participates, actually increases the aggressiveness of PDAC growth and metastasis while indirectly reducing desmoplasia [4]. More direct targeting of major ECM components, such as using a pegylated hyaluronidase (PEGPH20) to target hyaluronan, showed greatest promise in patients with advanced, unresectable PDAC when combined with gemcitabine and nab-paclitaxel; increasing patient survival in phase II trials to further warrant advancement into randomized phase III trials [5, 6]. Unfortunately, no discernable differences were observed between experimental and control arms, likely due to administration of low-dose PEGPH20 required to minimize major adverse effects associated with systemic hyaluronan degradation. In contrast to IPI-926, however, direct targeting of hyaluronan with PEGPH20 was shown to decrease metastases in an autochthonous PDAC model in combination with chemotherapy [7]. Similarly, direct targeting of collagen with collagenase-loaded nanoparticles has also been shown to increase efficacy of chemotherapy without increasing the metastatic potential of autochthonous PDAC tumors [8]. However, as with hyaluronidase, collagenase-based strategies have the potential to cause severe systemic toxicities due to the abundance of collagen in normal tissues.

The advent of immunotherapy has rekindled interest in microbial-based therapies for cancer, which are considered the first immunotherapies developed by William B. Coley in the late 1800s. Microbial-based platforms, such as attenuated *Salmonella typhimurium* (ST), have been utilized for their ability to colonize and replicate in solid tumors when administered systemically. In our most recent work, we were successful in engineering tumor-colonizing ST expressing functional hyaluronidase (ST-HAse) [9]. In contrast to previous methods, this agent specifically colonizes PDAC tumors, efficiently degrades hyaluronan, and improves chemotherapeutic delivery and efficacy. No degradation was observed in hyaluronan-rich tissues such as the skin and joints. Direct enzymatic degradation of hyaluronan using ST-HAse will most likely not change or decrease the incidence of metastasis based on previous studies using PEGHP20. Interestingly, we observed greater ST tumor diffusion following hyaluronan degradation, suggesting that ST-HAse treatment has the potential to enhance entry of relatively large agents including antibodies and cell-based therapies [9, 10]. In addition to bacteria, oncolytic viruses have also been engineered to encode hyaluronidases, but are dependent on infected tumor and stromal cells for expression, decreasing their efficacy [11]. Altogether, targeting tumor-associated ECM, while minimizing degradation in normal tissue, appears to be the most promising approach to improving therapeutic delivery in PDAC.

It is also worth noting that, in the era of immunotherapy, important roles for ECM components in suppressing anti-tumor responses have been uncovered. High- and low-molecular weight hyaluronans and tenascin C are known to signal through toll-like receptors and regulate inflammatory, angiogenic, fibrotic, and cancer promoting processes [12]. The accumulation of hyaluronan in the tumor ECM strongly correlates with progression by enhancing tumor cell proliferation, invasion, and metastasis [13]. In addition to hyaluronan, collagens expressed in the tumor ECM are known to act as ligands for the inhibitory receptor LAIR-1, which inhibits the function of multiple immune subsets including T and NK cells [14]. Indeed, depletion of these major ECM components has been shown to enhance immune infiltration [7, 15]. In conclusion, targeting desmoplasia will not only improve drug delivery in PDAC, but may also enhance efficacy of current immunotherapeutic approaches.
